# Supplementary material for: Does ‘When’ really feel more certain than ‘If’? Two failures to replicate Ballard and Lewandowsky (2015)
Source: R Soc Open Sci. 2019 Jul 10;6(7):180475. doi: 10.1098/rsos.180475 (PMC6689617; doi:10.1098/rsos.180475)
Supplement: Attention Filters and Programmed Survey for Experiment 1 [file rsos180475supp1.pdf]

Attention Checks Used in Experiment 1

(1) Recent research on decision-making shows that choices are affected by context. To help us understand how people make decisions, we are interested in information about you. Specifically, we're interested in whether you take the time to read the directions. If not, some results may not tell us very much about decision-making in the real world. To show that you have read the instructions, please ignore the question below about how you are feeling and instead check only "none of the above."

Please check all the words that describe how you are currently feeling:

Response options: Attentive, Nervous, Distressed, Optimistic, Skeptical, Scared, Hopeful, None of the Above

(2) Most research on scientific communication recognizes that decisions do not take place in a vacuum. Individual preferences and prior knowledge can influence how information is received and understood. To demonstrate that you're read this much, please select "prefer not to answer" to the question below.

How interested are you in learning more about the temperature increases and sea level rise associated with climate change?

Response options:
1-7; with labels at 1 (not at all interested), 3 (Mildly interested), 5 (Somewhat interested) and 7 (Very interested); There were also the options: Don't know and Prefer not to answer

(3) People are very busy these days and many do not have time to follow science news on climate change. Some do pay attention but do not read questions carefully. To show that you're read this much, please choose "other" below and type "k" into the "other" box.

How interested are you in following science news on climate change?

Response options:
Not at all interested
A little interested
Somewhat interested

Very interested
Other: ______

Attention Checks Used in Original Ballard and Lewandowsky (2015) Experiment

Temperature section

"Which of these is not an animal?"

Table
Dog
Cat
Mouse

Sea level section

"What is 2 + 3?"

2
3
4
5
6

Acidification section

"What is the second letter of the English alphabet?"

A
B
C
D

Arctic ice section

"Select 'none of the above' from the options below."

All of the above
Some of the above
None of the above

intro & consent form

Browser Meta Info

This question will not be displayed to the recipient.
Browser: **Chrome**
Version: **71.0.3578.98**
Operating System: **Windows NT 10.0**
Screen Resolution: **1920x1080**
Flash Version: **-1**
Java Support: **0**
User Agent: **Mozilla/5.0 (Windows NT 10.0; Win64; x64) AppleWebKit/537.36 (KHTML, like Gecko) Chrome/71.0.3578.98 Safari/537.36**

INTRODUCTION & CONSENT

RESEARCH PROCEDURES

This research is being conducted to help us understand people's interpretations of scientific information. If you agree to participate, you may be asked to read short informational statements and answer questions about them. You will also be asked questions about yourself (your age, gender, etc.), and your opinions. The survey takes 10 to 15 minutes to complete.

RISKS

There are no foreseeable risks for participating in this research.

BENEFITS

There are no benefits to you as a participant other than to further research on communicating information.

CONFIDENTIALITY

The data in this study will be confidential, and the researchers will not be able to identify you. The researchers will not have your name, address, or any other identifying information. If the results from this study are published, only summary results will be reported and individual responses will not be identifiable.

PARTICIPATION

Your participation is voluntary, and you may withdraw from the study at any time and for any reason.

If you decide not to participate or if you withdraw from the study, there is no penalty or loss of benefits to which you are otherwise entitled. There are no costs to you or any other party.

CONTACTS

This research is being conducted by Dr. Connie Roser-Renouf from the Department of Communication at George Mason University. She may be reached at (707) 832-9666 or at croserre@gmu.edu for questions or to report a research-related problem. You may contact the George Mason University Office of Research Integrity & Assurance at 703-993-4121 if you have questions or comments regarding your rights as a participant in the research.

This research has been reviewed according to George Mason University procedures governing your participation in research.

CONSENT

I have read this form, and I agree to participate in this study, realizing that I may withdraw at any time. I understand that all information that I provide is strictly confidential, and will not be released by the investigator in any form that may identify me.

Please indicate whether or not you are willing to participate in the study. Clicking the YES button below indicates that you have decided to participate.

- ○ No
- ○ [ ] Yes [Please date below]

Thank you for agreeing to participate in this study!

Block 1: uncertain outcome; certain time; distant projection

These page timer metrics will not be displayed to the recipient.

First Click: *0 seconds*

Last Click: *0 seconds*

#QuestionText, TimingPageSubmit#: *0 seconds*

#QuestionText, TimingClickCount#: *0 clicks*

TRAINING:

During this part of the survey, you will be asked to read two statements about climate change and to look at graphs that correspond to each statement. The statement below is an example of the type of statement you will be asked to read.

"Scientists project that by 2065, the population of emperor penguins will decrease to 1500 to 2500 breeding pairs."

The graph below corresponds to the above statement. It shows the estimated population of emperor penguins over the course of this century. The information in the surrounding boxes will help you interpret the data shown in the graph.

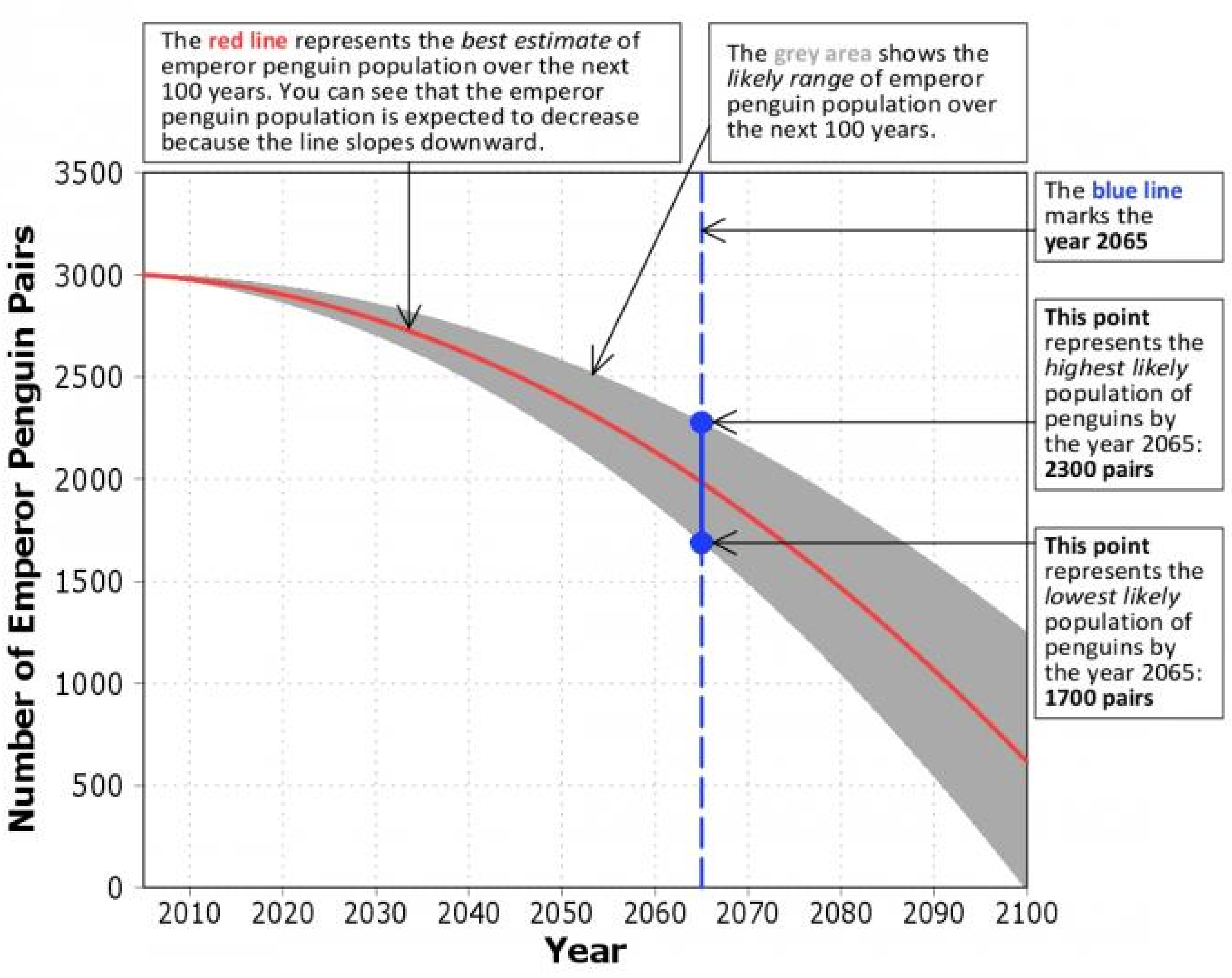

Based on the above information, how likely is it that there will be **1000** breeding emperor penguin pairs in the year **2090**?

- Unlikely
- Likely

Based on the above information, how likely is it that there will be **3000** breeding emperor penguin pairs in the year **2070**?

- Unlikely
- Likely

These page timer metrics will not be displayed to the recipient.
First Click: *0 seconds*
Last Click: *0 seconds*
#QuestionText, TimingPageSubmit#: *0 seconds*
#QuestionText, TimingClickCount#: *0 clicks*

Please read the following information carefully:

In the first part of this survey, you will receive some general information about the expected effects of climate change (sometimes referred to as global warming) on global temperatures and sea level. You will be shown the anticipated changes in temperatures and sea level and will be asked several questions about these projections. It is important to note that **all** of the information you will receive about climate risks and predictions is **accurate** and based on **cutting-edge climate science data**.

TEMPERATURE INCREASES

Global temperatures have increased by 1.4°F since the beginning of industrialization some 150 years ago. Rising temperatures have negative effects, such as increasing the frequency and intensity of summer heat waves. Warmer climates also contribute to the spread of diseases (such as malaria) and pests (such as pine bark beetles). Similarly, in most regions of the world, agricultural productivity is expected to decline owing to factors such as more prolonged droughts, decreased water supply, and increasing fire frequency. Paradoxically, agriculture will be under threat not only

from increased drought, but also from the greater intensity of rainfall—when it occurs—and hence flooding.

Now please read the following statement which provides information about the expected future change in global surface temperature due to climate change:

"Scientists project that by 2065, global average surface temperature will rise by 3.0 to 4.3 degrees Fahrenheit."

The graph below corresponds to the above statement. It shows the estimated global surface warming over the course of this century.

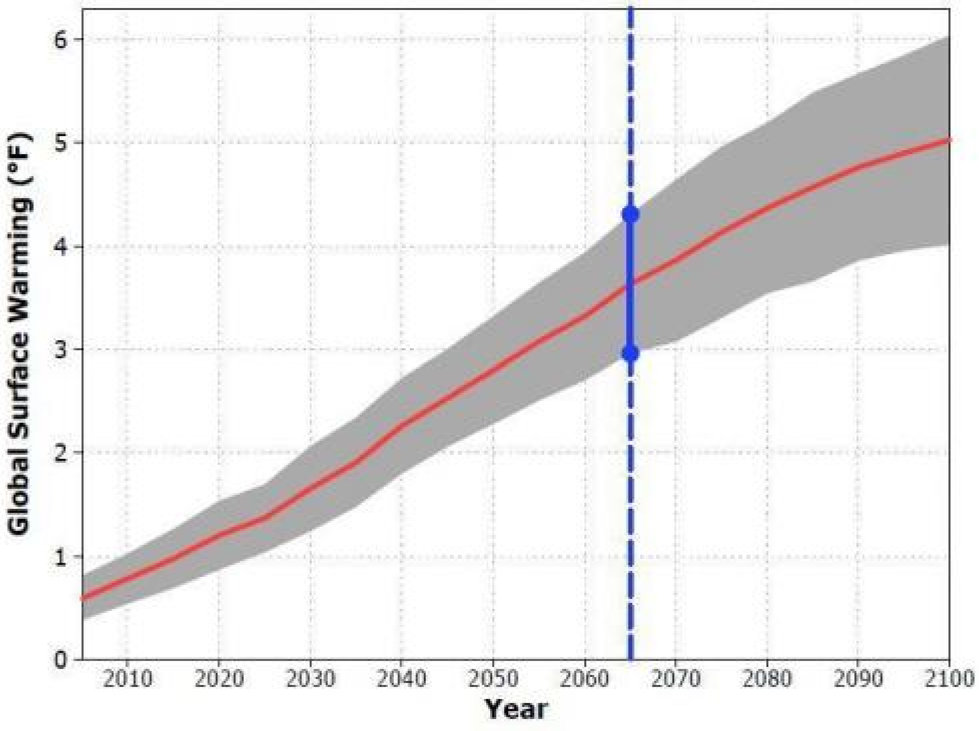

How clear is the information presented in the figure?

Completely unclear (1)	(2)	Somewhat unclear (3)	(4)	Neither clear nor unclear (5)	(6)	Somewhat clear (7)	(8)	Completely clear (9)
○	○	○	○	○	○	○	○	○

How much effort did it take for you to understand the figure?

No effort at all (1)	(2)	Very little effort (3)	(4)	Some effort (5)	(6)	A fair amount of effort (7)	(8)	A great deal of effort (9)	I did not understand the figure
○	○	○	○	○	○	○	○	○	○

Recent research on decision-making shows that choices are affected by context. To help us understand how people make decisions, we are interested in information about you. Specifically, we're interested in whether you take the time to read the directions. If not, some results may not tell us very much about decision-making in the real world. To show that you have read the instructions, please ignore the question below about how you are feeling and instead check only "none of the above."

Please check all the words that describe how you are currently feeling:

- ☐ Interested
- ☐ Upset
- ☐ Attentive
- ☐ Nervous
- ☐ Distressed
- ☐ Optimistic
- ☐ Angry
- ☐ Guilty
- ☐ Skeptical
- ☐ Scared
- ☐ Hopeful
- ☐ None of the above

These page timer metrics will not be displayed to the recipient.
First Click: *0 seconds*
Last Click: *0 seconds*
#QuestionText, TimingPageSubmit#: *0 seconds*
#QuestionText, TimingClickCount#: *0 clicks*

Here's the figure again. We have two more questions for you about it.

"Scientists project that by 2065, global average surface temperature will rise by 3.0 to 4.3 degrees Fahrenheit."

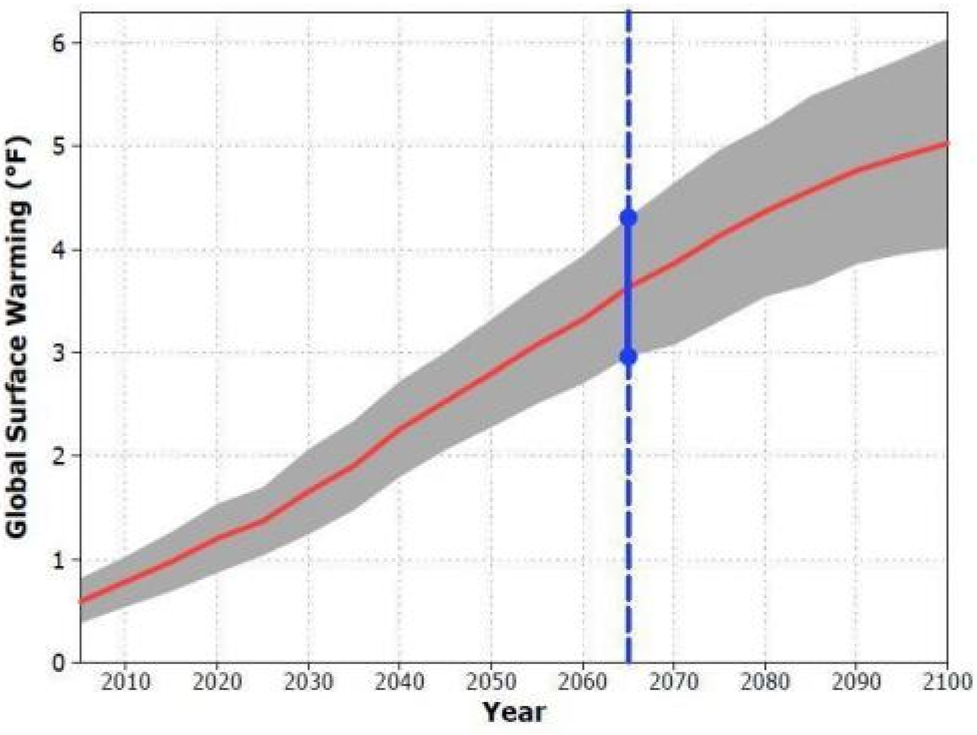

How certain are you that by 2065, average global surface temperature will rise by 3.0 to 4.3 degrees Fahrenheit?

Not at all certain (1)	(2)	Slightly certain (3)	(4)	Moderately certain (5)	(6)	Very certain (7)	(8)	Extremely certain (9)
○	○	○	○	○	○	○	○	○

How concerned are you by this projected amount of global warming?

Not at all concerned (1)	(2)	Slightly concerned (3)	(4)	Moderately concerned (5)	(6)	Very concerned (7)	(8)	Extremely concerned (9)
○	○	○	○	○	○	○	○	○

These page timer metrics will not be displayed to the recipient.

First Click: *0 seconds*

Last Click: *0 seconds*
#QuestionText, TimingPageSubmit#: *0 seconds*
#QuestionText, TimingClickCount#: *0 clicks*

SEA LEVEL RISE

A consequence of climate change will be rising sea levels around the world. As the world warms, the oceans absorb more heat and therefore expand. In addition, land ice from Greenland and Antarctica and most of the world's glaciers, is slowly melting. The runoff from melting land ice feeds into rivers and directly into the oceans. This too adds to rising sea levels. The combined effects of expanding volume and runoff have changed sea levels by 4 inches already since 1970. This amount is sufficient to cause coastal erosion and landslides in many places around the world. In addition, sea level rise also makes storm surges more damaging.

Now please read the following statement which provides information about the expected future change in sea level due to climate change:

"Scientists project that by 2065, sea level will rise by 12 to 23 inches."

The graph below corresponds to the above statement. It shows the estimated sea level change over the course of this century.

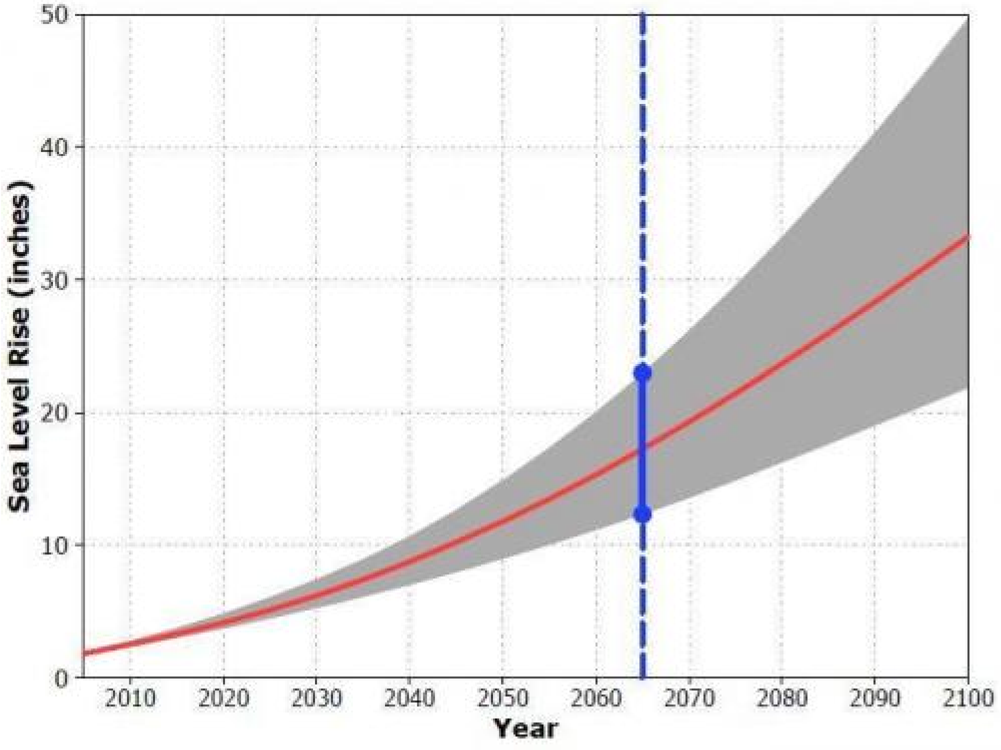

How clear is the information presented in the figure?

- Completely unclear (1)
- (2)
- Somewhat unclear (3)
- (4)
- Neither clear nor unclear (5)
- (6)
- Somewhat clear (7)
- (8)
- Completely clear (9)

How much effort did it take for you to understand the figure?

- No effort at all (1)
- (2)
- Very little effort (3)
- (4)
- Some effort (5)
- (6)
- A fair amount of effort (7)
- (8)
- A great deal of effort (9)
- I did not understand the figure

These page timer metrics will not be displayed to the recipient.

First Click: *0 seconds*

Last Click: *0 seconds*

#QuestionText, TimingPageSubmit#: *0 seconds*

#QuestionText, TimingClickCount#: *0 clicks*

Here's the figure again. We have three more questions for you about it.

"Scientists project that by 2065, sea level will rise by 12 to 23 inches."

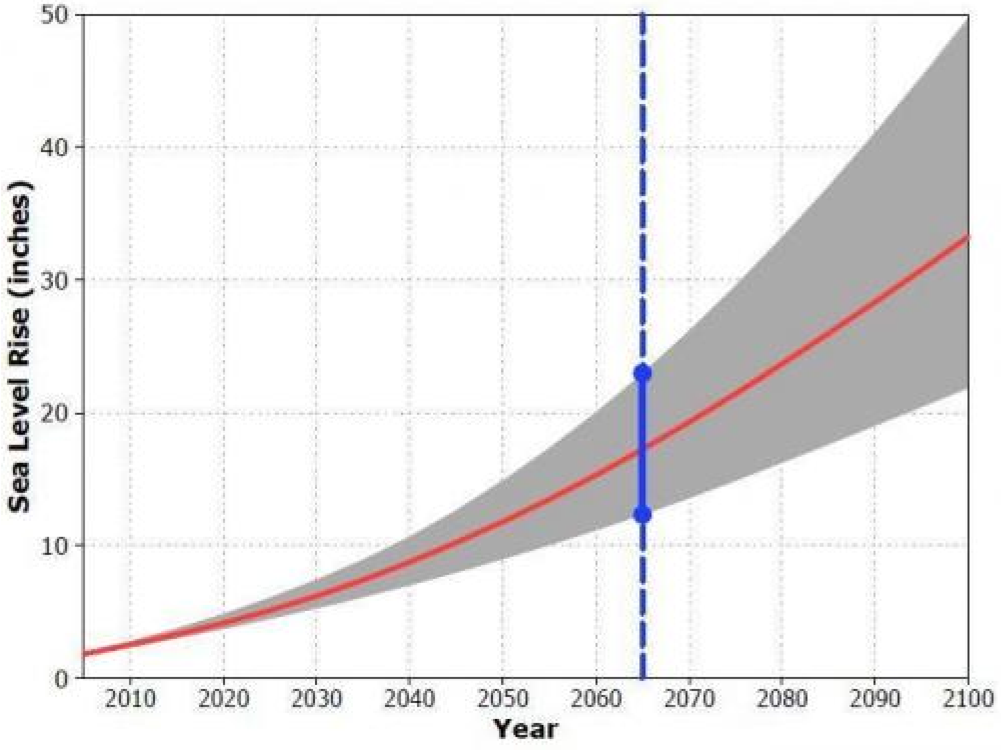

How certain are you that by 2065 sea level will rise by 12 to 23 inches?

Not at all certain (1)	(2)	Slightly certain (3)	(4)	Moderately certain (5)	(6)	Very certain (7)	(8)	Extremely certain (9)
○	○	○	○	○	○	○	○	○

How concerned are you by this projected amount of sea level rise?

Not at all concerned (1)	(2)	Slightly concerned (3)	(4)	Moderately concerned (5)	(6)	Very concerned (7)	(8)	Extremely concerned (9)
○	○	○	○	○	○	○	○	○

Most research on scientific communication recognizes that decisions do not take place in a vacuum. Individual preferences and prior knowledge can influence how information is received and understood. To demonstrate that you're read this much, please select "prefer not to answer" to the question below.

How interested are you in learning more about the temperature increases and sea level rise associated with climate change?

Not at all interested (1)	(2)	Mildly interested (3)	(4)	Somewhat interested (5)	(6)	Very interested (7)	Don't know (98)	Prefer not to answer (99)
○	○	○	○	○	○	○	○	○

Now we'd like to ask some questions about you, your opinions, and your beliefs.

Block 2: certain outcome; uncertain time; distant projection

These page timer metrics will not be displayed to the recipient.
First Click: *0 seconds*
Last Click: *0 seconds*
#QuestionText, TimingPageSubmit#: *0 seconds*
#QuestionText, TimingClickCount#: *0 clicks*

TRAINING:

During this part of the survey, you will be asked to read two statements about climate change and to look at graphs that correspond to each statement. The statement below is an example of the type of statement you will be asked to read.

"Scientists project that the population of emperor penguins will decrease to 2000 breeding pairs between 2050 and 2080."

The graph below corresponds to the above statement. It shows the estimated population of emperor penguins over the course of this century. The information in the surrounding boxes will help you interpret the data shown in the graph.

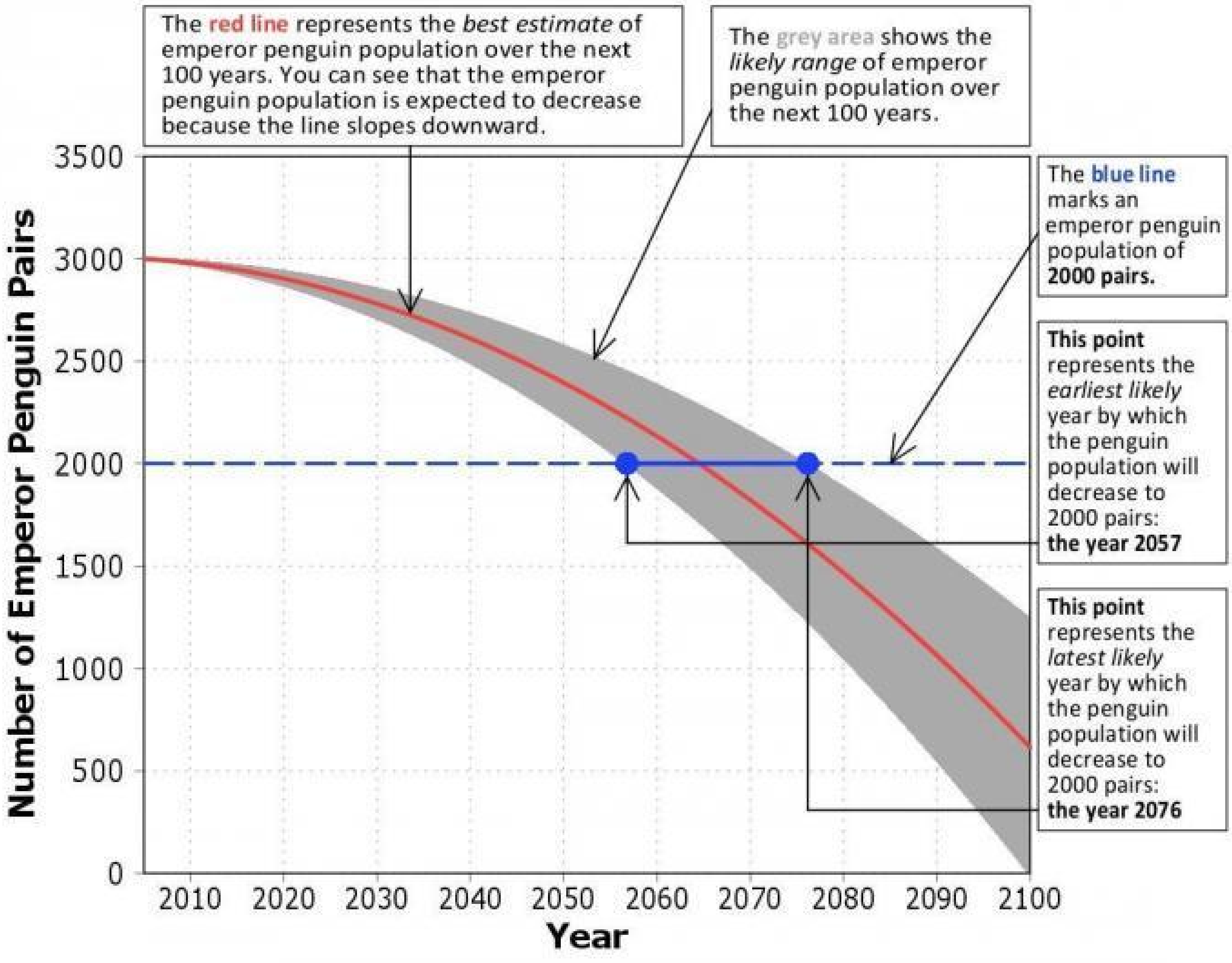

Based on the above information, how likely is it that there will be **1000** breeding emperor penguin pairs in the year **2090**?

- Unlikely
- Likely

Based on the above information, how likely is it that there will be **3000** breeding emperor penguin pairs in the year **2070**?

- Unlikely
- Likely

These page timer metrics will not be displayed to the recipient.
First Click: *0 seconds*
Last Click: *0 seconds*
#QuestionText, TimingPageSubmit#: *0 seconds*
#QuestionText, TimingClickCount#: *0 clicks*

Please read the following information carefully:

In the first part of this survey, you will receive some general information about the expected effects of climate change (sometimes referred to as global warming) on global temperatures and sea level. You will be shown the anticipated changes in temperatures and sea level and will be asked several questions about the projections. It is important to note that **all** of the information you will receive about climate risks and predictions is **accurate** and based on **cutting-edge climate science data**.

TEMPERATURE INCREASES

Global temperatures have increased by 1.4°F since the beginning of industrialization some 150 years ago. Rising temperatures have negative effects, such as increasing the frequency and intensity of summer heat waves. Warmer climates also contribute to the spread of diseases (such as malaria) and pests (such as pine bark beetles). Similarly, in most regions of the world, agricultural productivity is expected to decline owing to factors such as more prolonged droughts, decreased water supply, and increasing fire frequency. Paradoxically, agriculture will be under threat not only from increased drought, but also from the greater intensity of rainfall—when it occurs—and hence flooding.

Now please read the following statement which provides information about the expected future change in global surface temperature due to climate change:

"Scientists project that global average surface temperature will increase by 3.6 degrees Fahrenheit between 2054 and 2083."

The graph below corresponds to the above statement. It shows the estimated global surface warming over the course of this century.

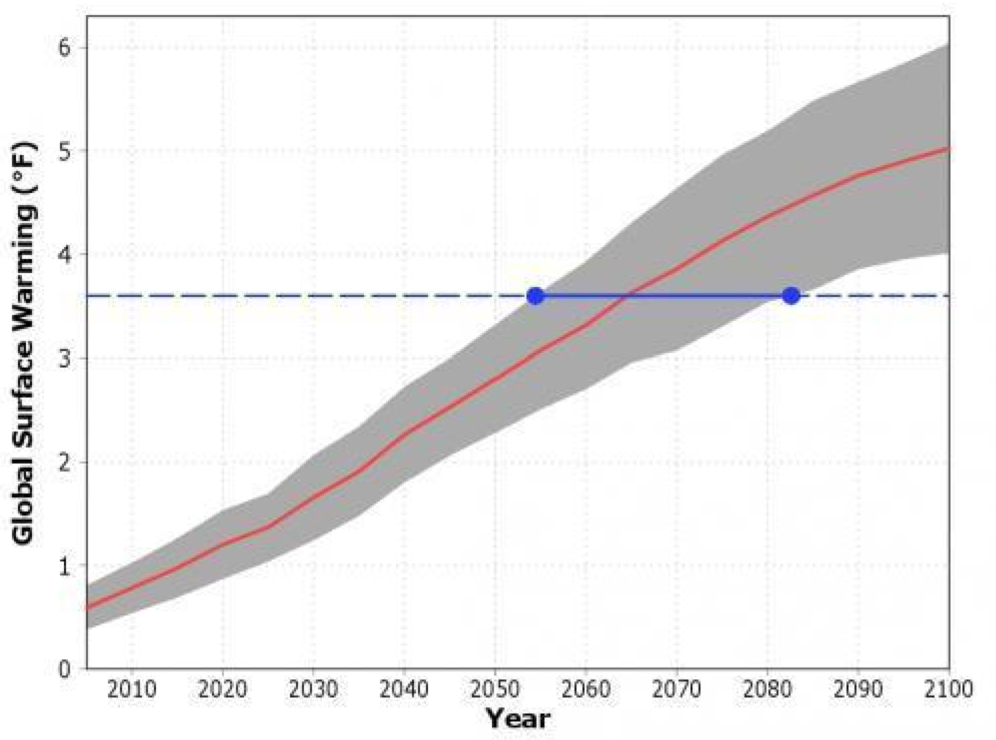

How clear is the information presented in the figure?

Completely unclear (1) | (2) | Somewhat unclear (3) | (4) | Neither clear nor unclear (5) | (6) | Somewhat clear (7) | (8) | Completely clear (9)

How much effort did it take for you to understand the figure?

No effort at all (1) | (2) | Very little effort (3) | (4) | Some effort (5) | (6) | A fair amount of effort (7) | (8) | A great deal of effort (9) | I did not understand the figure

Recent research on decision-making shows that choices are affected by context. To help us understand how people make decisions, we are interested in information

about you. Specifically, we're interested in whether you take the time to read the directions. If not, some results may not tell us very much about decision-making in the real world. To show that you have read the instructions, please ignore the question below about how you are feeling and instead check only "none of the above."

Please check all the words that describe how you are currently feeling:

- ☐ Interested
- ☐ Upset
- ☐ Attentive
- ☐ Nervous
- ☐ Distressed
- ☐ Optimistic
- ☐ Angry
- ☐ Guilty
- ☐ Skeptical
- ☐ Scared
- ☐ Hopeful
- ☐ None of the above

These page timer metrics will not be displayed to the recipient.

First Click: *0 seconds*

Last Click: *0 seconds*

#QuestionText, TimingPageSubmit#: *0 seconds*

#QuestionText, TimingClickCount#: *0 clicks*

Here's the figure again. We have two more questions for you about it.

"Scientists project that global average surface temperature will increase by 3.6 degrees Fahrenheit between 2054 and 2083."

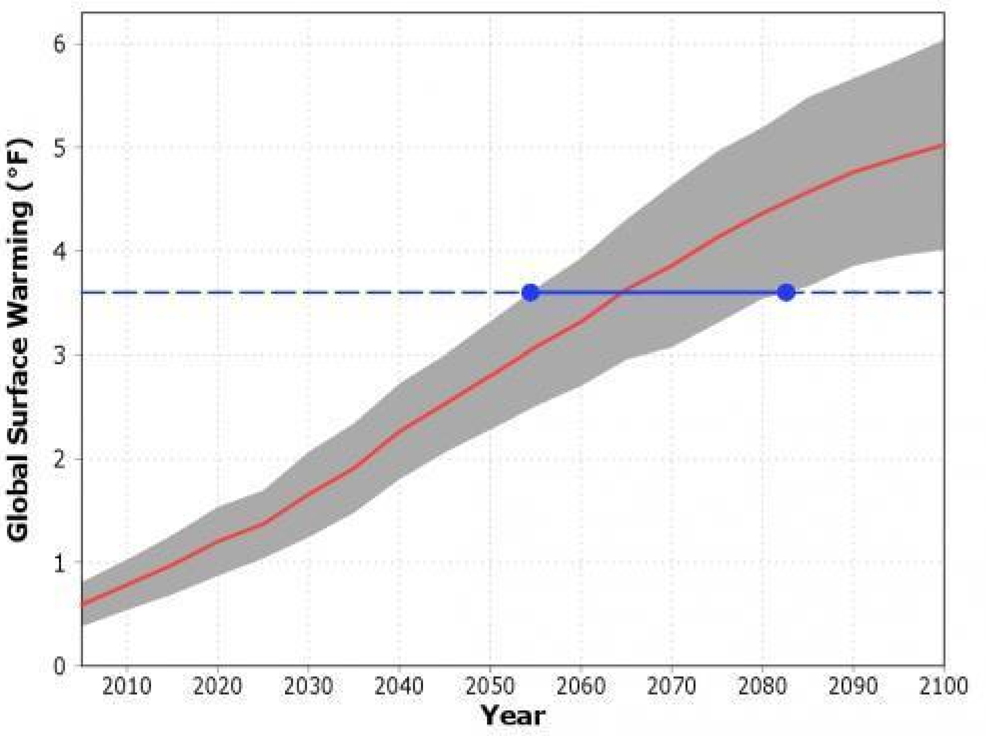

How certain are you global average surface temperature with increase by 3.6 degrees Fahrenheit between 2054 and 2083?

Not at all certain (1)	(2)	Slightly certain (3)	(4)	Moderately certain (5)	(6)	Very certain (7)	(8)	Extremely certain (9)
○	○	○	○	○	○	○	○	○

How concerned are you by this projected amount of global warming?

Not at all concerned (1)	(2)	Slightly concerned (3)	(4)	Moderately concerned (5)	(6)	Very concerned (7)	(8)	Extremely concerned (9)
○	○	○	○	○	○	○	○	○

These page timer metrics will not be displayed to the recipient.

First Click: *0 seconds*

Last Click: *0 seconds*

#QuestionText, TimingPageSubmit#: *0 seconds*

#QuestionText, TimingClickCount#: *0 clicks*

SEA LEVEL RISE

A consequence of climate change will be rising sea levels around the world. As the world warms, the oceans absorb more heat and therefore expand. In addition, land ice from Greenland and Antarctica and most of the world's glaciers, is slowly melting. The runoff from melting land ice feeds into rivers and directly into the oceans. This too adds to rising sea levels. The combined effects of expanding volume and runoff have changed sea levels by 4 inches already since 1970. This amount is sufficient to cause coastal erosion and landslides in many places around the world. In addition, sea level rise also makes storm surges more damaging.

Now please read the following statement which provides information about the expected future change in sea level due to climate change:

"Scientists predict that sea levels will rise by 17 inches between 2055 and 2084."

The graph below corresponds to the above statement. It shows the estimated sea level change over the course of this century.

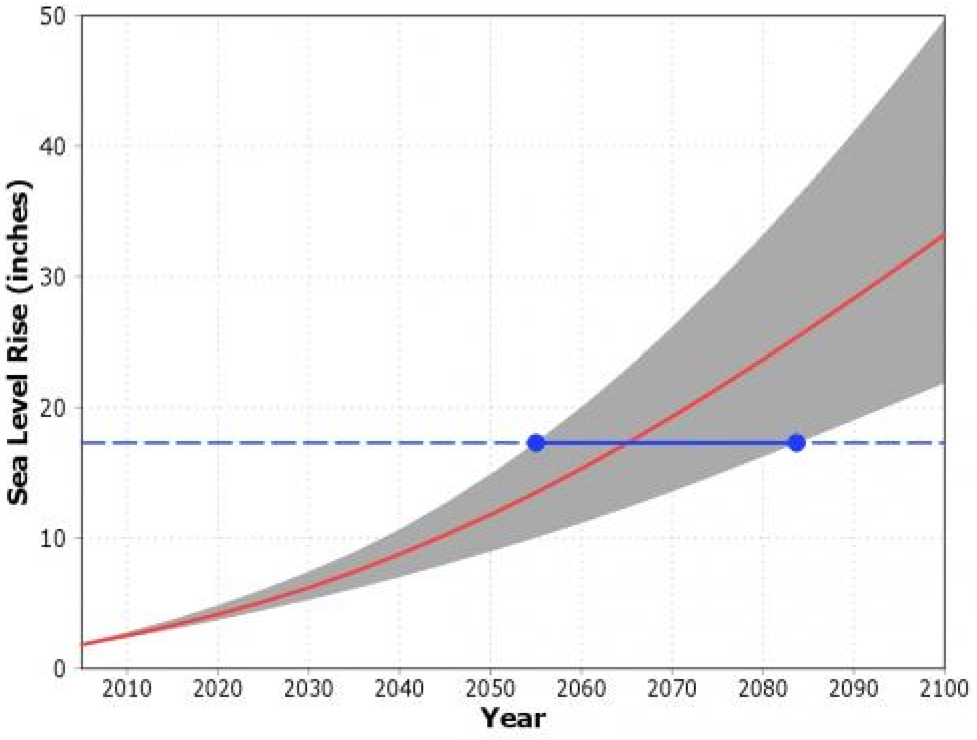

How clear is the information presented in the figure?

Completely unclear (1)	(2)	Somewhat unclear (3)	(4)	Neither clear nor unclear (5)	(6)	Somewhat clear (7)	(8)	Completely clear (9)
○	○	○	○	○	○	○	○	○

How much effort did it take for you to understand the figure?

No effort at all (1)	(2)	Very little effort (3)	(4)	Some effort (5)	(6)	A fair amount of effort (7)	(8)	A great deal of effort (9)	I did not understand the figure
○	○	○	○	○	○	○	○	○	○

These page timer metrics will not be displayed to the recipient.

First Click: *0 seconds*

Last Click: *0 seconds*

#QuestionText, TimingPageSubmit#: *0 seconds*

#QuestionText, TimingClickCount#: *0 clicks*

Here's the figure again. We have three more questions for you about it.

"Scientists predict that sea levels will rise by 17 inches between 2055 and 2084."

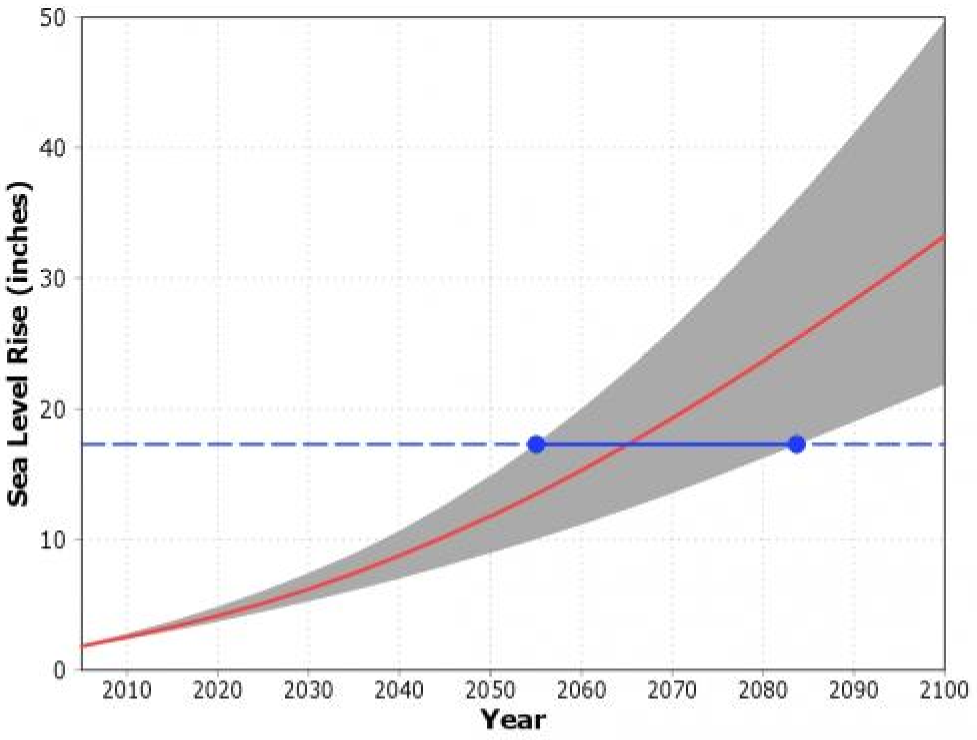

How certain are you that sea level will rise by 17 inches between 2055 and 2084?

- Not at all certain (1)
- (2)
- Slightly certain (3)
- (4)
- Moderately certain (5)
- (6)
- Very certain (7)
- (8)
- Extremely certain (9)

How concerned are you by this projected amount of sea level rise?

- Not at all concerned (1)
- (2)
- Slightly concerned (3)
- (4)
- Moderately concerned (5)
- (6)
- Very concerned (7)
- (8)
- Extremely concerned (9)

Most research on scientific communication recognizes that decisions do not take place in a vacuum. Individual preferences and prior knowledge can influence how information is received and understood. To demonstrate that you're read this much, please select "prefer not to answer" to the question below.

How interested are you in learning more about the temperature increases and sea level rise associated with global warming?

Not at all interested (1)	(2)	Mildly interested (3)	(4)	Somewhat interested (5)	(6)	Very interested (7)	Don't know (98)	Prefer not to answer (99)
○	○	○	○	○	○	○	○	○

Now we'd like to ask some questions about you, your opinions, and your beliefs.

Block 3: uncertain outcome, certain time, proximal projection

These page timer metrics will not be displayed to the recipient.
First Click: *0 seconds*
Last Click: *0 seconds*
#QuestionText, TimingPageSubmit#: *0 seconds*
#QuestionText, TimingClickCount#: *0 clicks*

TRAINING:

During this part of the survey, you will be asked to read two statements about climate change and to look at graphs that correspond to each statement. The statement below is an example of the type of statement you will be asked to read.

"Scientists project that by 2065, the population of emperor penguins will decrease to 1500 to 2500 breeding pairs."

The graph below corresponds to the above statement. It shows the estimated population of emperor penguins over the course of this century. The information in the surrounding boxes will help you interpret the data shown in the graph.

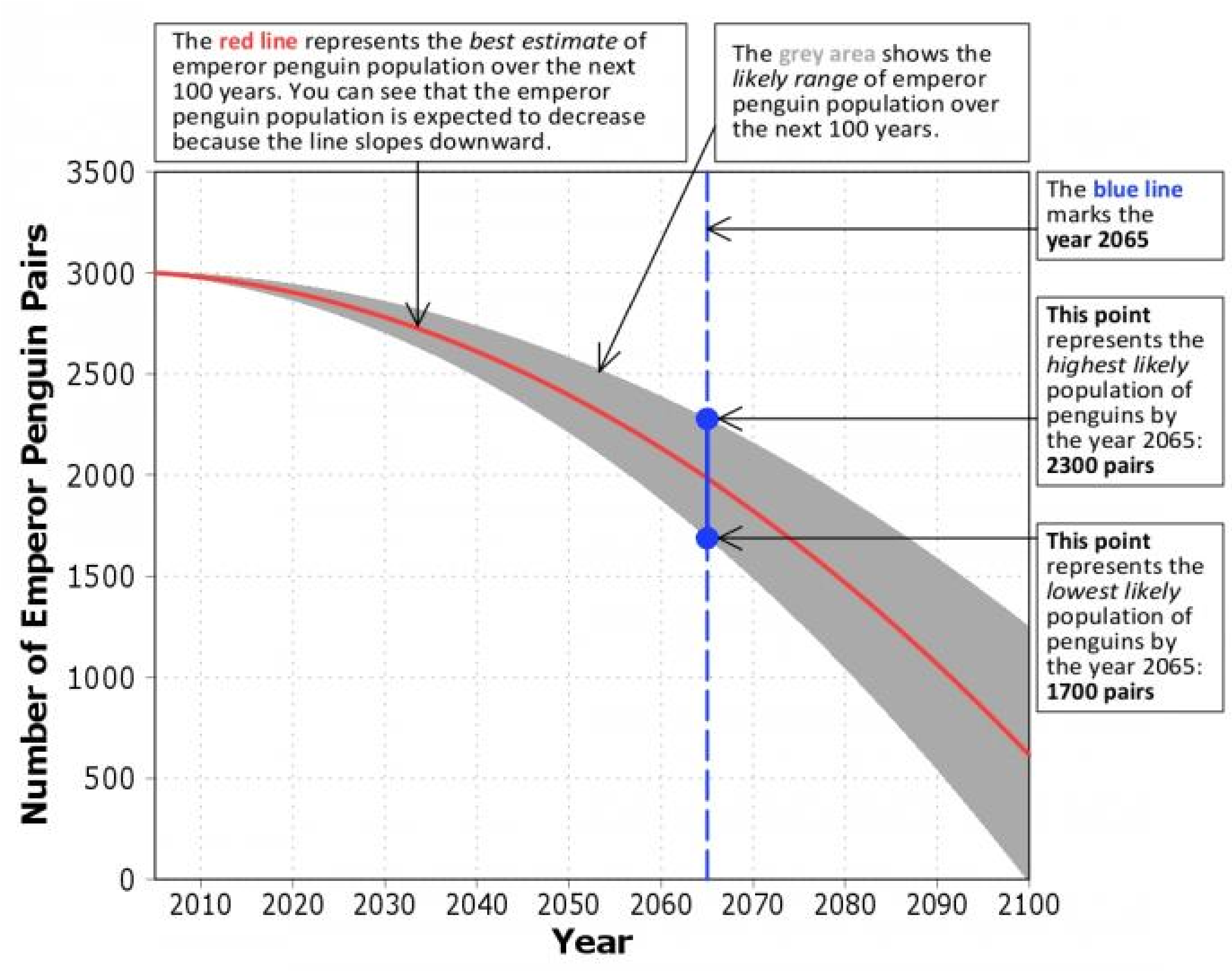

Based on the above information, how likely is it that there will be **1000** breeding emperor penguin pairs in
the year **2090**?

- Unlikely
- Likely

Based on the above information, how likely is it that there will be **3000** breeding emperor penguin pairs in the year **2070**?

- Unlikely
- Likely

These page timer metrics will not be displayed to the recipient.
First Click: *0 seconds*
Last Click: *0 seconds*
#QuestionText, TimingPageSubmit#: *0 seconds*
#QuestionText, TimingClickCount#: *0 clicks*

Please read the following information carefully:

In the first part of this survey, you will receive some general information about the expected effects of climate change (sometimes referred to as global warming) on global temperatures and sea level. You will be shown the anticipated changes in temperatures and sea level and will be asked several questions about the projections. It is important to note that **all** of the information you will receive about climate risks and predictions is **accurate** and based on **cutting-edge climate science data**.

TEMPERATURE INCREASES

Global temperatures have increased by 1.4°F since the beginning of industrialization some 150 years ago. Rising temperatures have negative effects, such as increasing the frequency and intensity of summer heat waves. Warmer climates also contribute to the spread of diseases (such as malaria) and pests (such as pine bark beetles). Similarly, in most regions of the world, agricultural productivity is expected to decline owing to factors such as more prolonged droughts, decreased water supply, and increasing fire frequency. Paradoxically, agriculture will be under threat not only from increased drought, but also from the greater intensity of rainfall—when it occurs—and hence flooding.

Now please read the following statement which provides information about the expected future change in global surface temperature due to climate change:

"Scientists project that by 2035, global average surface temperatures will increase by 1.5 to 2.3 degrees Fahrenheit."

The graph below corresponds to the above statement. It shows the estimated global surface warming over the course of this century.

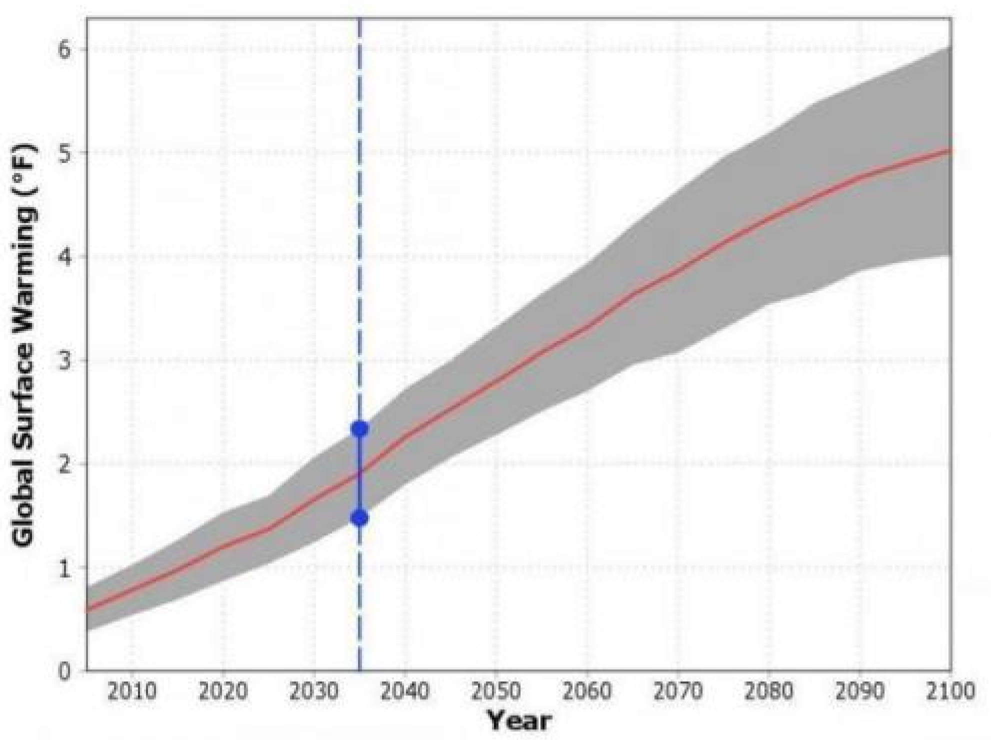

How clear is the information presented in the figure?

Completely unclear (1)	(2)	Somewhat unclear (3)	(4)	Neither clear nor unclear (5)	(6)	Somewhat clear (7)	(8)	Completely clear (9)
O	O	O	O	O	O	O	O	O

How much effort did it take for you to understand the figure?

No effort at all (1)	(2)	Very little effort (3)	(4)	Some effort (5)	(6)	A fair amount of effort (7)	(8)	A great deal of effort (9)	I did not understand the figure
O	O	O	O	O	O	O	O	O	O

Recent research on decision-making shows that choices are affected by context. To help us understand how people make decisions, we are interested in information about you. Specifically, we're interested in whether you take the time to read the

directions. If not, some results may not tell us very much about decision-making in the real world. To show that you have read the instructions, please ignore the question below about how you are feeling and instead check only "none of the above."

Please check all the words that describe how you are currently feeling:

- ☐ Interested
- ☐ Upset
- ☐ Attentive
- ☐ Nervous
- ☐ Distressed
- ☐ Optimistic
- ☐ Angry
- ☐ Guilty
- ☐ Skeptical
- ☐ Scared
- ☐ Hopeful
- ☐ None of the above

These page timer metrics will not be displayed to the recipient.
First Click: *0 seconds*
Last Click: *0 seconds*
#QuestionText, TimingPageSubmit#: *0 seconds*
#QuestionText, TimingClickCount#: *0 clicks*

Here's the figure again. We have two more questions for you about it.

"Scientists project that by 2035, global average surface temperatures will increase by 1.5 to 2.3 degrees Fahrenheit."

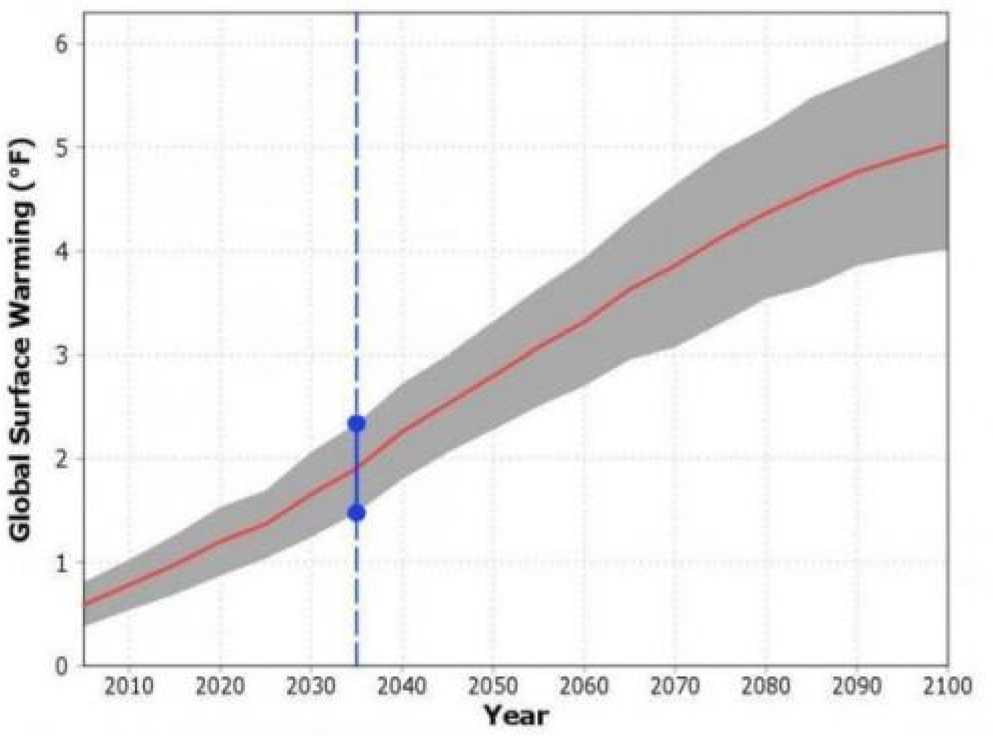

How certain are you that by 2035, average global surface temperature will rise by 1.5 to 2.3 degrees Fahrenheit?

Not at all certain (1)	(2)	Slightly certain (3)	(4)	Moderately certain (5)	(6)	Very certain (7)	(8)	Extremely certain (9)
○	○	○	○	○	○	○	○	○

How concerned are you by this projected amount of global warming?

Not at all concerned (1)	(2)	Slightly concerned (3)	(4)	Moderately concerned (5)	(6)	Very concerned (7)	(8)	Extremely concerned (9)
○	○	○	○	○	○	○	○	○

These page timer metrics will not be displayed to the recipient.

First Click: *0 seconds*

Last Click: *0 seconds*

#QuestionText, TimingPageSubmit#: *0 seconds*

#QuestionText, TimingClickCount#: *0 clicks*

SEA LEVEL RISE

A consequence of climate change will be rising sea levels around the world. As the world warms, the oceans absorb more heat and therefore expand. In addition, land ice from Greenland and Antarctica and most of the world's glaciers, is slowly melting. The runoff from melting land ice feeds into rivers and directly into the oceans. This too adds to rising sea levels. The combined effects of expanding volume and runoff have changed sea levels by 4 inches already since 1970. This amount is sufficient to cause coastal erosion and landslides in many places around the world. In addition, sea level rise also makes storm surges more damaging.

Now please read the following statement which provides information about the expected future change in sea level due to climate change:

"Scientists project that by 2035, sea level will rise by 6 to 9 inches."

The graph below corresponds to the above statement. It shows the estimated sea level change over the course of this century.

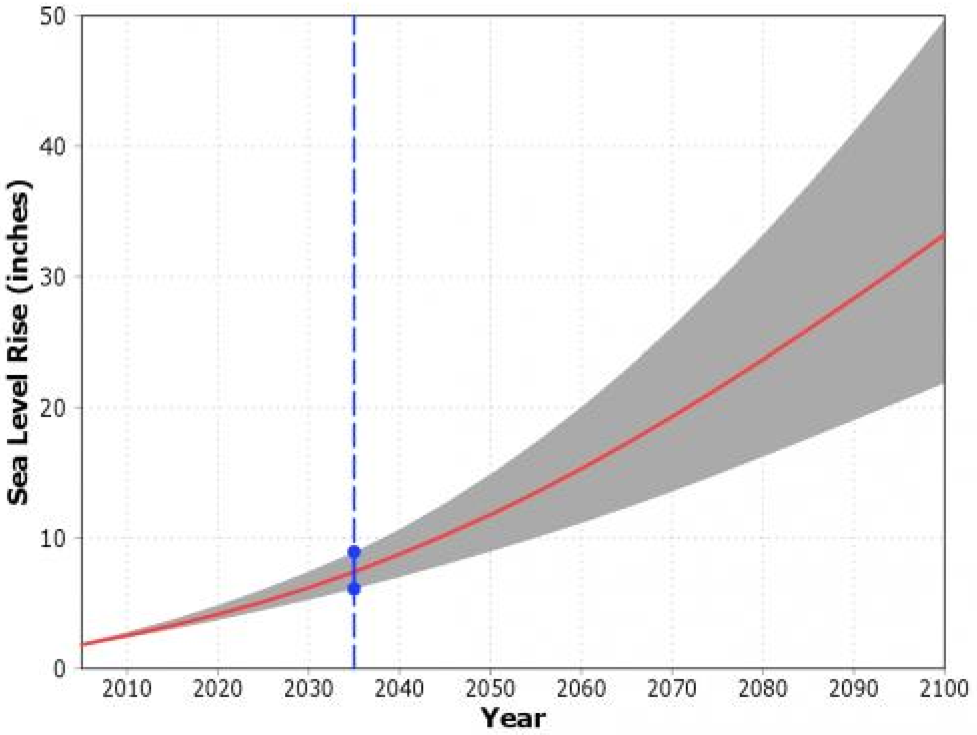

How clear is the information presented in the figure?

Completely unclear (1)	(2)	Somewhat unclear (3)	Click to write Choice (4)	Neither clear nor unclear (5)	(6)	Somewhat clear (7)	(8)	Completely clear (9)
○	○	○	○	○	○	○	○	○

How much effort did it take for you to understand the figure?

No effort at all (1)	(2)	Very little effort (3)	(4)	Some effort (5)	(6)	A fair amount of effort (7)	(8)	A great deal of effort (9)	I did not understand the figure
○	○	○	○	○	○	○	○	○	○

These page timer metrics will not be displayed to the recipient.

First Click: *0 seconds*

Last Click: *0 seconds*

#QuestionText, TimingPageSubmit#: *0 seconds*

#QuestionText, TimingClickCount#: *0 clicks*

Here's the figure again. We have three more questions for you about it.

"Scientists project that by 2035, sea level will rise by 6 to 9 inches."

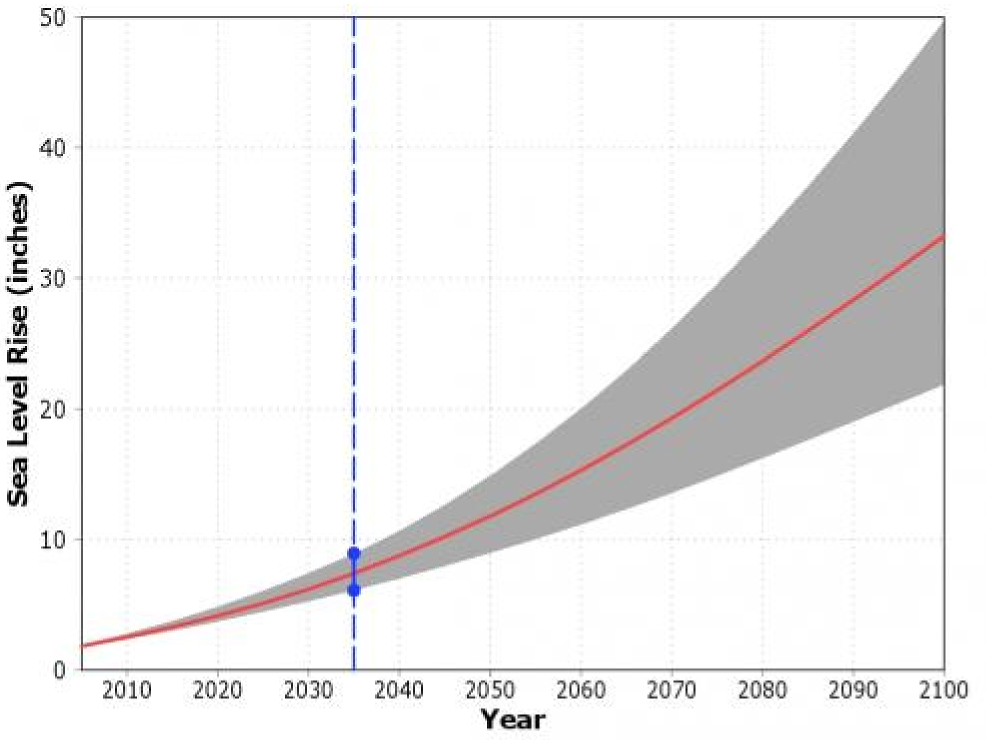

How certain are you that by 2035 sea level will rise by 6 to 9 inches?

Not at all certain (1)	(2)	Slightly certain (3)	(4)	Moderately certain (5)	(6)	Very certain (7)	(8)	Extremely certain (9)
○	○	○	○	○	○	○	○	○

How concerned are you by this projected amount of sea level rise?

Not at all concerned (1)	(2)	Slightly concerned (3)	(4)	Moderately concerned (5)	(6)	Very concerned (7)	(8)	Extremely concerned (9)
○	○	○	○	○	○	○	○	○

Most research on scientific communication recognizes that decisions do not take place in a vacuum. Individual preferences and prior knowledge can influence how information is received and understood. To demonstrate that you're read this much, please select "prefer not to answer" to the question below.

How interested are you in learning more about the temperature increases and sea level rise associated with global warming?

Not at all interested (1)	(2)	Mildly interested (3)	(4)	Somewhat interested (5)	(6)	Very interested (7)	Don't know (98)	Prefer not to answer (99)
○	○	○	○	○	○	○	○	○

Now we'd like to ask some questions about you, your opinions, and your beliefs.

Block 4: certain outcome; uncertain time; proximal projection

These page timer metrics will not be displayed to the recipient.
First Click: *0 seconds*
Last Click: *0 seconds*
#QuestionText, TimingPageSubmit#: *0 seconds*
#QuestionText, TimingClickCount#: *0 clicks*

TRAINING

During this part of the survey, you will be asked to read two statements about climate change and to look at graphs that correspond to each statement. The statement below is an example of the type of statement you will be asked to read.

"Scientists project that the population of emperor penguins will decrease to 2000 breeding pairs between 2050 and 2080."

The graph below corresponds to the above statement. It shows the estimated population of emperor penguins over the course of this century. The information in the surrounding boxes will help you interpret the data shown in the graph.

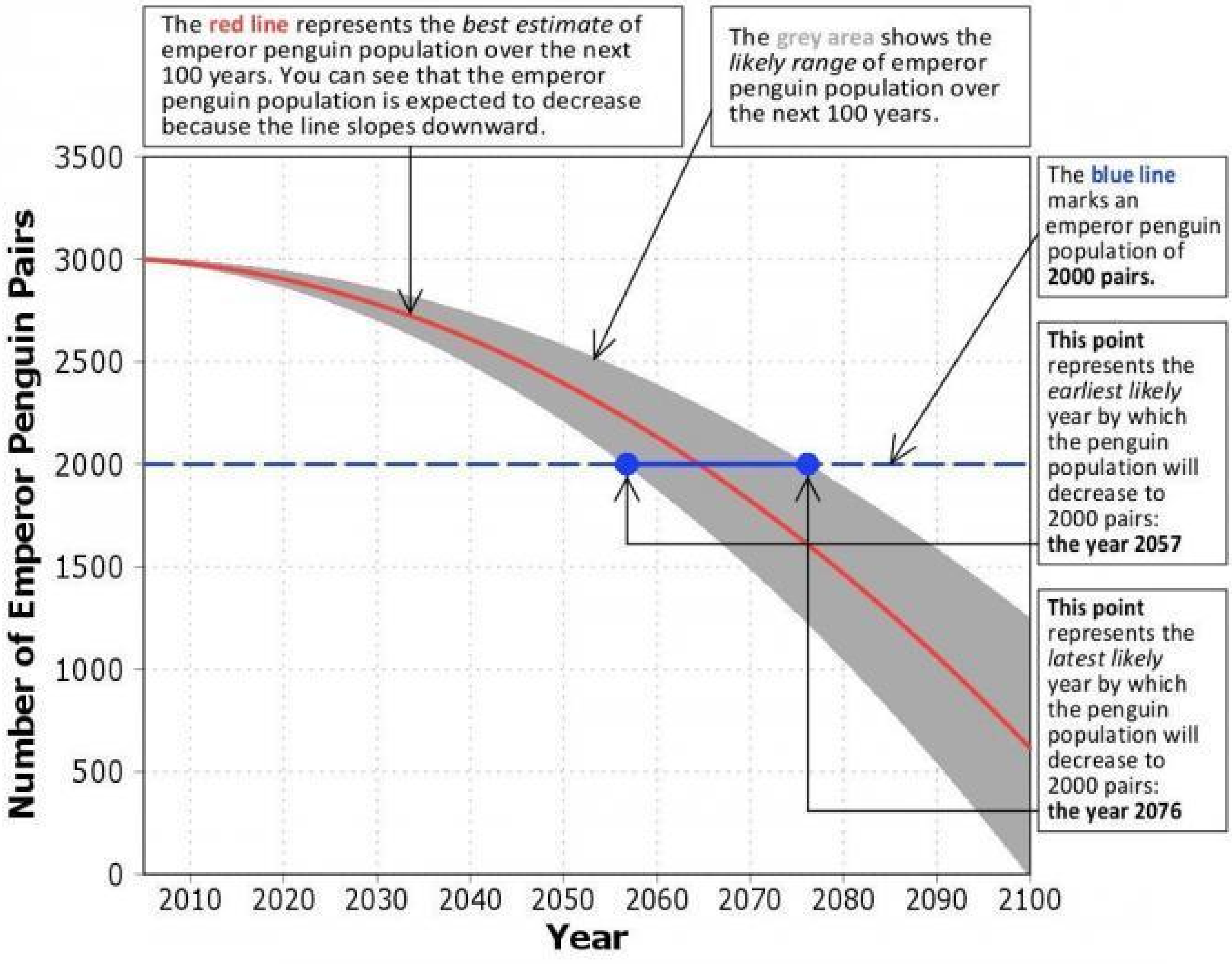

Based on the above information, how likely is it that there will be **1000** breeding emperor penguin pairs in the year **2090**?

- Unlikely
- Likely

Based on the above information, how likely is it that there will be **3000** breeding emperor penguin pairs in the year **2070**?

- Unlikely
- Likely

These page timer metrics will not be displayed to the recipient.
First Click: *0 seconds*
Last Click: *0 seconds*
#QuestionText, TimingPageSubmit#: *0 seconds*
#QuestionText, TimingClickCount#: *0 clicks*

Please read the following information carefully:

In the first part of this survey, you will receive some general information about the expected effects of climate change (sometimes referred to as global warming) on global temperatures and sea level. You will be shown the anticipated changes in temperatures and sea level and will be asked several questions about the projections. It is important to note that **all** of the information you will receive about climate risks and predictions is **accurate** and based on **cutting-edge climate science data**.

TEMPERATURE INCREASES

Global temperatures have increased by 1.4°F since the beginning of industrialization some 150 years ago. Rising temperatures have negative effects, such as increasing the frequency and intensity of summer heat waves. Warmer climates also contribute to the spread of diseases (such as malaria) and pests (such as pine bark beetles). Similarly, in most regions of the world, agricultural productivity is expected to decline owing to factors such as more prolonged droughts, decreased water supply, and increasing fire frequency. Paradoxically, agriculture will be under threat not only from increased drought, but also from the greater intensity of rainfall—when it occurs—and hence flooding.

Now please read the following statement which provides information about the expected future change in global surface temperature due to climate change:

"Scientists project that global average surface temperature will increase by 1.9 degrees Fahrenheit between 2028 and 2042."

The graph below corresponds to the above statement. It shows the estimated global surface warming over the course of this century.

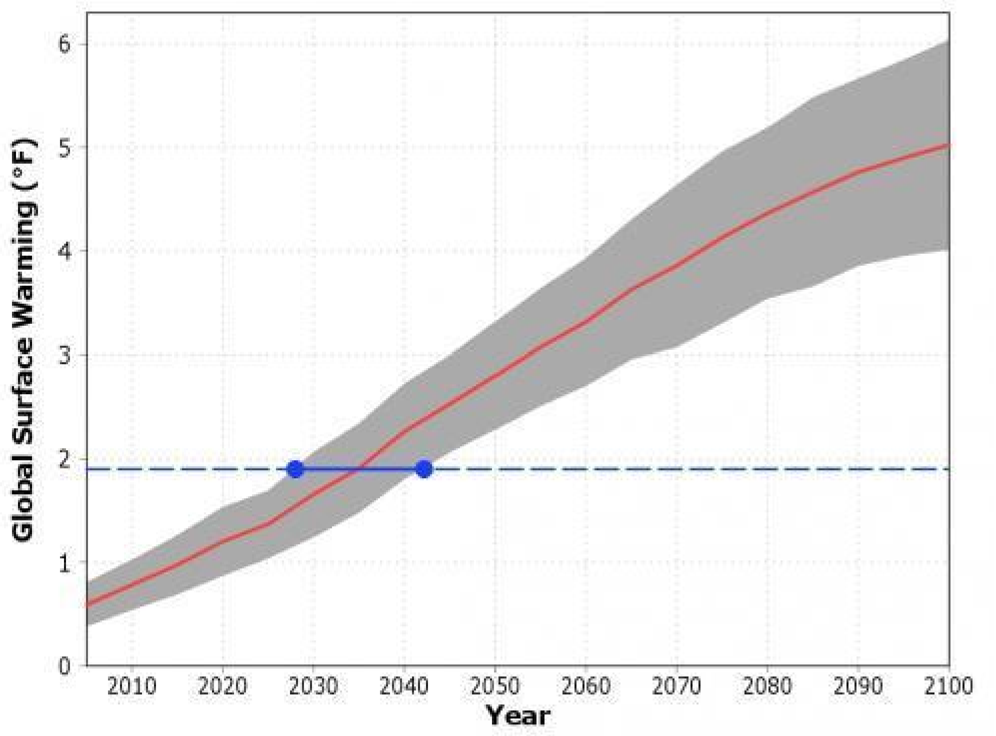

How clear is the information presented in the figure?

Completely unclear (1)	(2)	Somewhat unclear (3)	(4)	Neither clear nor unclear (5)	(6)	Somewhat clear (7)	(8)	Completely clear (9)
○	○	○	○	○	○	○	○	○

How much effort did it take for you to understand the figure?

No effort at all (1)	(2)	Very little effort (3)	(4)	Some effort (5)	(6)	A fair amount of effort (7)	(8)	A great deal of effort (9)	I did not understand the figure
○	○	○	○	○	○	○	○	○	○

Recent research on decision-making shows that choices are affected by context. To help us understand how people make decisions, we are interested in information about you. Specifically, we're interested in whether you take the time to read the directions. If not, some results may not tell us very much about decision-making in

the real world. To show that you have read the instructions, please ignore the question below about how you are feeling and instead check only "none of the above."

Please check all the words that describe how you are currently feeling:

- ☐ Interested
- ☐ Upset
- ☐ Attentive
- ☐ Nervous
- ☐ Distressed
- ☐ Optimistic
- ☐ Angry
- ☐ Guilty
- ☐ Skeptical
- ☐ Scared
- ☐ Hopeful
- ☐ None of the above

These page timer metrics will not be displayed to the recipient.

First Click: *0 seconds*

Last Click: *0 seconds*

#QuestionText, TimingPageSubmit#: *0 seconds*

#QuestionText, TimingClickCount#: *0 clicks*

Here's the figure again. We have two more questions for you about it.

"Scientists project that global average surface temperature will increase by 1.9 degrees Fahrenheit between 2028 and 2042."

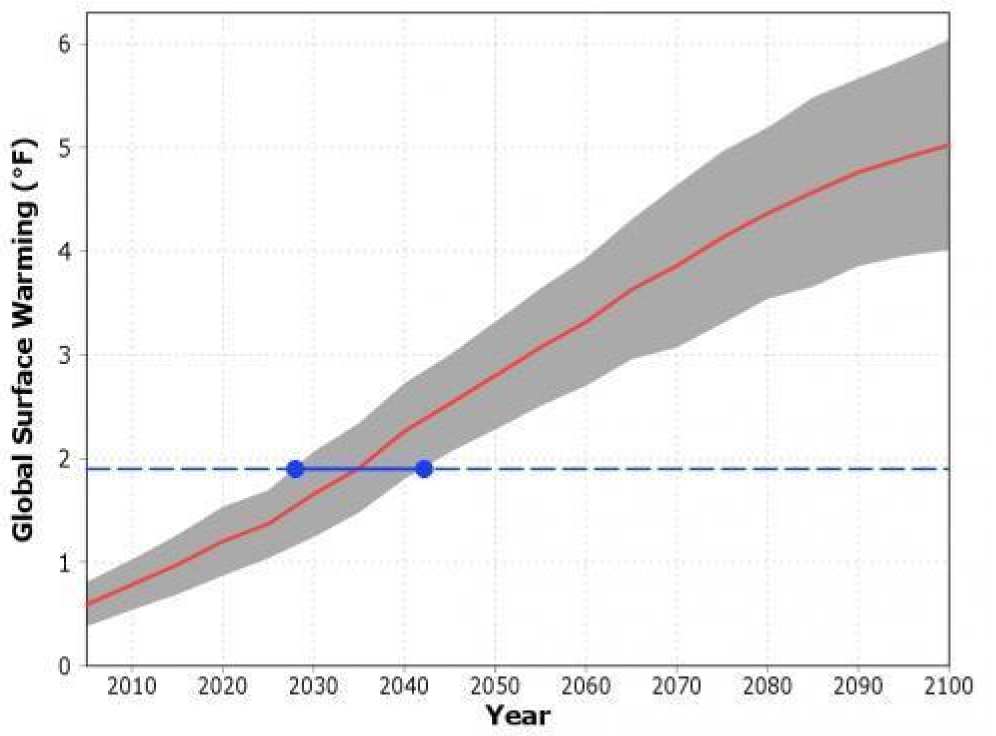

How certain are you that global average surface temperature with increase by 1.9 degrees Fahrenheit between 2028 and 2042?

Not at all certain (1)	(2)	Slightly certain (3)	(4)	Moderately certain (5)	(6)	Very certain (7)	(8)	Extremely certain (9)
○	○	○	○	○	○	○	○	○

How concerned are you by this projected amount of global warming?

Not at all concerned (1)	(2)	Slightly concerned (3)	(4)	Moderately concerned (5)	(6)	Very concerned (7)	(8)	Extremely concerned (9)
○	○	○	○	○	○	○	○	○

These page timer metrics will not be displayed to the recipient.

First Click: *0 seconds*

Last Click: *0 seconds*

#QuestionText, TimingPageSubmit#: *0 seconds*

#QuestionText, TimingClickCount#: *0 clicks*

SEA LEVEL RISE

A consequence of climate change will be rising sea levels around the world. As the world warms, the oceans absorb more heat and therefore expand. In addition, land ice from Greenland and Antarctica and most of the world's glaciers, is slowly melting. The runoff from melting land ice feeds into rivers and directly into the oceans. This too adds to rising sea levels. The combined effects of expanding volume and runoff have changed sea levels by 4 inches already since 1970. This amount is sufficient to cause coastal erosion and landslides in many places around the world. In addition, sea level rise also makes storm surges more damaging.

Now please read the following statement which provides information about the expected future change
in sea level due to climate change:

"Scientists project that sea levels will rise by 7 inches between 2030 and 2042."

The graph below corresponds to the above statement. It shows the estimated sea level change over the course of this century.

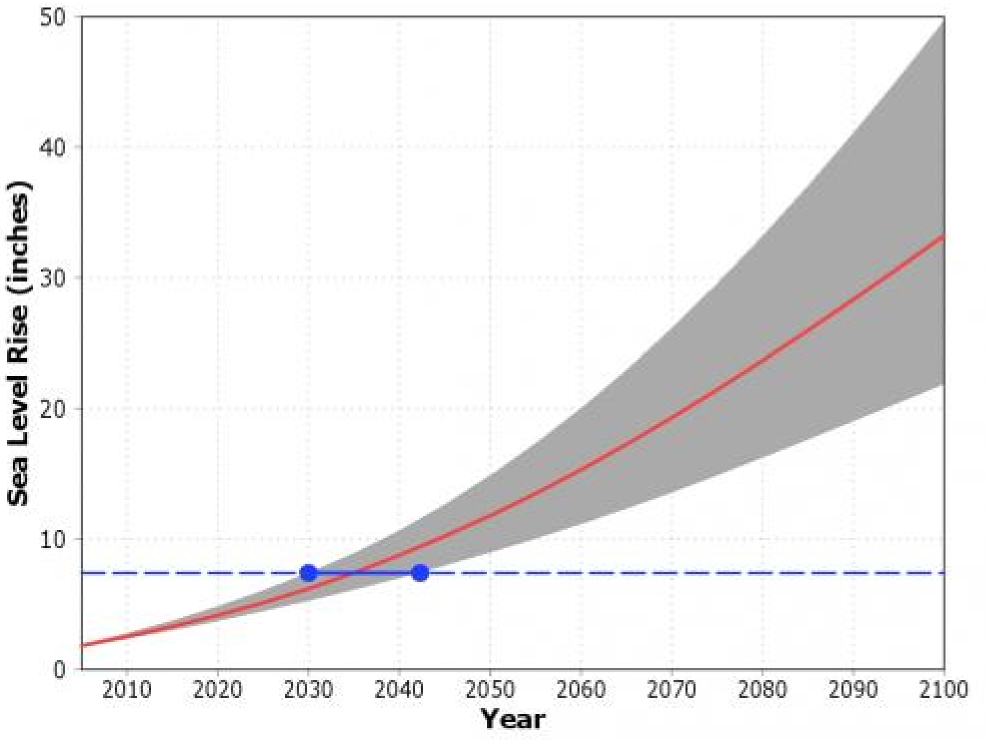

How clear is the information presented in the figure?

Completely unclear (1)	(2)	Somewhat unclear (3)	Click to write Choice (4)	Neither clear nor unclear (5)	(6)	Somewhat clear (7)	(8)	Completely clear (9)
○	○	○	○	○	○	○	○	○

How much effort did it take for you to understand the figure?

No effort at all (1)	(2)	Very little effort (3)	(4)	Some effort (5)	(6)	A fair amount of effort (7)	(8)	A great deal of effort (9)	I did not understand the figure
○	○	○	○	○	○	○	○	○	○

These page timer metrics will not be displayed to the recipient.

First Click: *0 seconds*

Last Click: *0 seconds*

#QuestionText, TimingPageSubmit#: *0 seconds*

#QuestionText, TimingClickCount#: *0 clicks*

Here's the figure again. We have three more questions for you about it.

"Scientists project that sea levels will rise by 7 inches between 2030 and 2042."

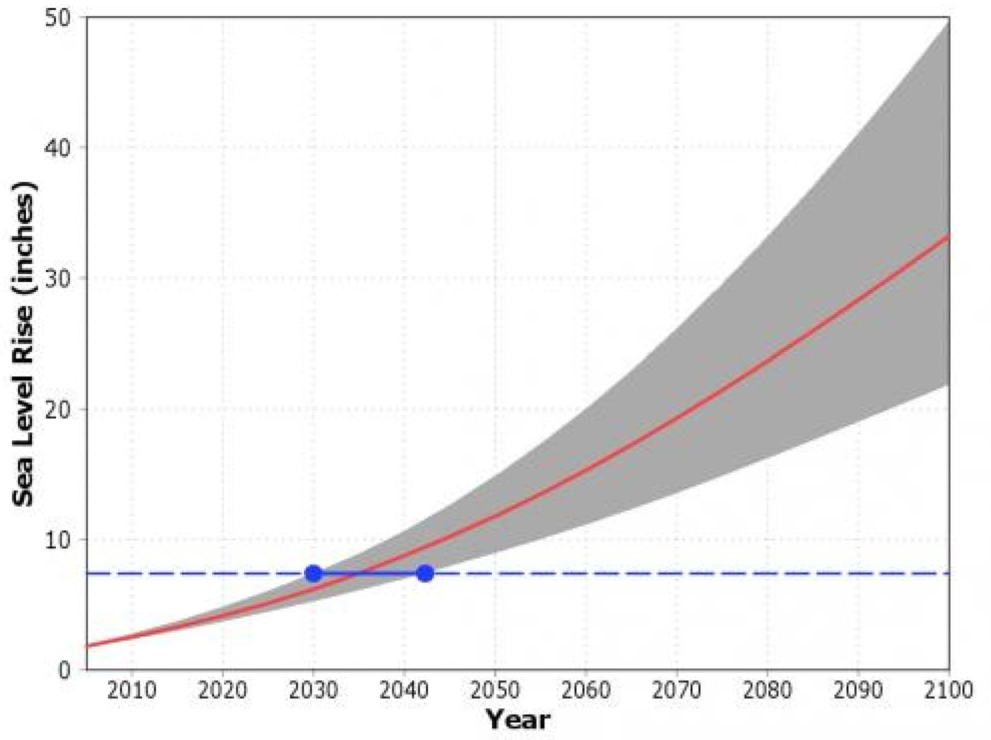

How certain are you that by 2065 sea level will rise by 7 inches between 2030 and 2042?

Not at all certain (1)	(2)	Slightly certain (3)	(4)	Moderately certain (5)	(6)	Very certain (7)	(8)	Extremely certain (9)
○	○	○	○	○	○	○	○	○

How concerned are you by the above projection?

Not at all concerned (1)	(2)	Slightly concerned (3)	(4)	Moderately concerned (5)	(6)	Very concerned (7)	(8)	Extremely concerned (9)
○	○	○	○	○	○	○	○	○

Most research on scientific communication recognizes that decisions do not take place in a vacuum. Individual preferences and prior knowledge can influence how information is received and understood. To demonstrate that you're read this much, please select "prefer not to answer" to the question below.

How interested are you in learning more about the temperature increases and sea level rise associated with global warming?

Not at all interested (1)	(2)	Mildly interested (3)	(4)	Somewhat interested (5)	(6)	Very interested (7)	Don't know (98)	Prefer not to answer (99)
○	○	○	○	○	○	○	○	○

Now we'd like to ask some questions about you, your opinions, and your beliefs.

Block 5: control group

We'd like to ask some questions about you, your opinions, and your beliefs.

Block 6: post-test

Do you think climate change is happening?

- ○ Yes
- ○ No
- ○ Don't know

How sure are you that climate change is happening?

- ○ Not at all sure
- ○ Somewhat sure
- ○ Very sure
- ○ Extremely sure

How sure are you that climate change is not happening?

- ○ Not at all sure

- Somewhat sure
- Very sure
- Extremely sure

Assuming climate change is happening, do you think it is…

- Caused entirely by human activities
- Caused mostly by human activities
- Caused about equally by human activities and natural changes in the environment
- Caused mostly by natural changes in the environment
- Caused entirely by natural changes in the environment
- None of the above because climate change isn't happening

Which comes closest to your view?

- Most scientists think climate change is not happening
- There is a lot of disagreement among scientists about whether or not climate change is happening
- Most scientists think climate change is happening
- Don't know enough to say

How certain are you that global surface temperatures ***will*** or ***will not*** increase over the next 100 years, if nothing is done to reduce climate change?

- Extremely certain global surface temperatures will increase
- Very certain global surface temperatures will increase
- Somewhat certain global surface temperatures will increase
- Don't know
- Somewhat certain global surface temperatures will ***not*** increase
- Very certain global surface temperatures will ***not*** increase

- Extremely certain global surface temperatures will ***not*** increase

How certain are you that sea level ***will*** or ***will not*** rise over the next 100 years, if nothing is done to reduce climate change?

- Extremely certain sea levels will rise
- Very certain sea levels will rise
- Somewhat certain sea levels will rise
- Don't know
- Somewhat certain sea levels will ***not*** rise
- Very certain sea levels will ***not*** rise
- Extremely certain sea levels will ***not*** rise

How worried are you about climate change?

- Very worried
- Somewhat worried
- Not very worried
- Not at all worried

How much do you think climate change will harm:

	Not at all	Only a little	A moderate amount	A great deal	Don't know
You personally	○	○	○	○	○
Your family	○	○	○	○	○
People in your community	○	○	○	○	○
People in the United States	○	○	○	○	○
People in other modern industrialized countries	○	○	○	○	○
People in developing countries	○	○	○	○	○

	Not at all	Only a little	A moderate amount	A great deal	Don't know
Future generations of people	○	○	○	○	○
Plant and animal species	○	○	○	○	○

When do you think climate change will start to harm people in the United States?

- They are being harmed now
- In 10 years
- In 25 years
- In 100 years
- Never

Which of the following statements comes closest to your view?

- Humans can reduce climate change, and we are going to do so successfully
- Humans could reduce climate change, but it's unclear at this point whether we'll do what's needed
- Humans could reduce climate change, but people aren't willing to change what they do, so we're not going to.
- Humans can't reduce climate change, even if it is happening
- Climate change isn't happening

How much had you thought about climate change before today?

- Not at all
- A little
- Some
- A lot

People are very busy these days and many do not have time to follow science news on climate change. Some do pay attention but do not read questions carefully. To show that you're read this much, please choose "other" below and type "k" into the "other" box.
How interested are you in following science news on climate change?

- Not at all interested
- A little interested
- Somewhat interested
- Very interested
- [ ] Other:

On some issues, people feel that they have all the information they need in order to form a firm opinion, while on other issues they would like more information before making up their mind. For climate change, where would you place yourself?

- I do not need any more information
- I need a little more information
- I need some more information
- I need a lot more information

People disagree whether the United States should reduce greenhouse gas emissions on its own, or make reductions only if other countries do so. Which of the following statements comes closest to your own point of view?

The United States should reduce its greenhouse gas emissions…

- Regardless of what other countries do
- Only if other industrialized nations (such as England, Germany and Japan) reduce their emissions
- Only if other industrialized nations and developing nations (such as India, China and Brazil) reduce their emissions
- The U.S. should not reduce its emissions

○ Don't know

How big of an effort should the United States make to reduce climate change?

○ A large-scale effort, even if it has large economic costs
○ A medium-scale effort, even if it has moderate economic costs
○ A small-scale effort, even if it has minor economic costs
○ No effort

Do you think the following should be doing more or less to *reduce climate change?*

	Much More	More	About the same	Less	Much less	Don't know
The U.S. Congress	○	○	○	○	○	○
President Obama	○	○	○	○	○	○
Your governor	○	○	○	○	○	○
Corporations and industry	○	○	○	○	○	○
Citizens themselves	○	○	○	○	○	○

Do you think each of the following should be doing more or less to *protect people from the impacts of climate change,* such as flooding, drought, and heat waves?

	Much less	Less	About the same	More	Much more	Don't know
The U.S. Congress	○	○	○	○	○	○
President Obama	○	○	○	○	○	○
Your governor	○	○	○	○	○	○
Your local government	○	○	○	○	○	○

In the past year have you personally experienced any of the extreme weather events or natural disasters listed below? Check all that apply.

☐ ☐

- Extreme snowstorm
- ☐ Extreme cold temperatures
- ☐ Extreme high winds
- ☐ Extreme rainstorm
- ☐ Extreme heat wave
- ☐ Extreme drought
- Flooding
- ☐ Hurricane
- ☐ Wildfire
- ☐ Tornado
- ☐ Earthquake
- ☐ None of the above

How much were you harmed by these events?

	Not at all (1)	(2)	Only a little (3)	(4)	A moderate amount (5)	(6)	A great deal (7)
Extreme snowstorm	○	○	○	○	○	○	○
Extreme cold temperatures	○	○	○	○	○	○	○
Extreme high winds	○	○	○	○	○	○	○
Extreme rainstorm	○	○	○	○	○	○	○
Extreme heat wave	○	○	○	○	○	○	○
Extreme drought	○	○	○	○	○	○	○
Flooding	○	○	○	○	○	○	○
Hurricane	○	○	○	○	○	○	○
Wildfire	○	○	○	○	○	○	○
Tornado	○	○	○	○	○	○	○
Earthquake	○	○	○	○	○	○	○

Generally speaking, do you think of yourself as a…

- ○ Republican
- ○ Democrat
- ○ Independent
- ○ Other
- ○ No party - not interested in politics

Do you think of yourself as closer to...

- The Republican party
- The Democratic party
- Neither

In general, do you think of yourself as...

- Very liberal
- Somewhat liberal
- Moderate, middle-of-the-road
- Somewhat conservative
- Very conservative

Are you...

- Male
- Female
- Other

What is your age? Please type in the number

What is the highest level of education you have completed?

- Less than High School
- High School / GED
- Some College
- 2-year College Degree
- 4-year College Degree

- Masters Degree
- Doctoral Degree
- Professional Degree (JD, MD)

Please indicate your marital status:

▼

What is your combined annual household income?

- Less than 30,000
- 30,000 – 39,999
- 40,000 – 49,999
- 50,000 – 59,999
- 60,000 – 69,999
- 70,000 – 79,999
- 80,000 – 89,999
- 90,000 – 99,999
- 100,000 or more

What is your race/ethnicity?

- Non-Hispanic White/Caucasian
- Non-Hispanic African-American
- Hispanic
- Asian
- Native American
- Pacific Islander
- Other

In which state do you currently reside?

Thank you very much for taking the time to complete this survey!

Powered by Qualtrics
